# Molecular docking based screening of a simulated HIF-1 protein model for potential inhibitors

**DOI:** 10.6026/97320630013388

**Published:** 2017-11-30

**Authors:** Mundla Sri Latha, Madhu Sudhana Saddala

**Affiliations:** 1 Centre for agricultural Bioinformatics, ICAR-IASRI (Indian Agricultural Statistics Research Institute), Library Avenue, Pusa, New Delhi - 110012, India;; 2 Johns Hopkins University, Wilmer's Eye Institute, School of Medicine, Baltimore, Maryland;; 3 Department of Biotechnology, Sri Venkateswara University, Tirupati - 517502, A.P., India;

**Keywords:** HIF-1, Homology modeling, docking, Zinc database, MD simulations, Chalcone

## Abstract

Hypoxia inducible factor-1(HIF-1) is a bHLH-family transcription factor that control genes involved in glucolysis, angiogenesis, migration, as well as invasion factors that are important for tumor progression and metastasis. HIF-1, a hetero dimer of HIF-1α and HIF-1β, binds to the hypoxia responsive genes, such as vascular endothelial growth factor (VEGF). It is one the molecular target for angiogenesis. A series of Chalcone - like compounds described that preferentially inhibit HIF-1 dimer, which can interact with amino acids within the active site of the protein. It is of interest model the HIF-1 dimer protein and protein was subjected to molecular dynamics simulations using NAMD 2.9 software with CHARMM27 force field in water and the protein structure was minimized with 25000 steps for 500 ps and simulation with 1000000 steps for 2ns. 2500 compounds were screened from Zinc database through structure based virtual screening with references to Chalcone natural drug compound. The screened compounds were docked into the active site of the protein using AutoDock Vina in PyRx Virtual screening tool. The docking result showed the compounds Zinc04280532, Zinc04280533, Zinc04280469, Zinc04280534, Zinc16405915, Zinc04277060, Zinc04280538, Zinc04582923, Zinc05280554 and Zinc05943723 have high binding affinities then query compound. The lead hit compounds were also testing for toxicity and bioavailability using Osiris and Molinspiration online server. The active site amino acids such as TYR-21, ASN-34, VAL-35, MET-18, LYS-17, SER-36, ARG- 46 and ARG-14 are key role in the inhibitors activity. This is useful in the design of small molecule therapeutics or the treatment of different abnormalities associated with impaired HIF-1α.

## Background

Angiogenesis is the physiological process through which new blood vessels form from pre-existing vessels. This is distinct from vasculogenesis, which is the de novo formation of endothelial cells [1] from mesoderm cell precursors. The first vessels in the embryo form through vasculogenesis, after which angiogenesis is responsible for most, if not all, blood vessel growth during development [2] and in disease. A hypoxic tumor occurs due to the increased metabolic rate and oxygen consumption of rapidly proliferating tumor cells [3]. The hypoxiaresponsive pathway allows tumor cells to overcome harsh conditions. The most important mediator identified in this pathway is hypoxia inducible factor-1 (HIF-1), a transcription factor for various angiogenic factors such as vascular endothelial growth factor (VEGF), and for genes encoding proteins involved in energy metabolism, cell survival, red blood cell production, and vasomotor regulation [4]. HIF-1 is a heterodimer consisting of HIF-11 and HIF-12 subunits. HIF-2 is a nuclear protein, whereas HIF-11 shuttles between the cytoplasm and nucleus [5]. The 1 and 1 subunits both belong to the basic helix-loop-helix (bHLH) PER-ARNT-SIM (PAS) domain family of transcription factors. In HIF-11, the N-terminal (bHLHPAS) domain is required for dimerization and DNA binding, whereas the C-terminal domains are required for hypoxia-induced nuclear localization, protein stabilization and transactivation [6, 7]. HIF-11 is stable only under hypoxia, and the accumulation of HIF-11 is followed by its entry into the nucleus, where HIF-11 binds with HIF-12. The two subunits then bind with a specific five-nucleotide DNA sequence (5'-RCGTG-3'), known as the hypoxia responsive element (HRE), located in the promoter regions of hypoxia-responsive genes [7]. The HIF-1 dimer binds to the HRE sequence (5'-TACGTG-3') in the VEGF promoter and induces the expression of VEGF. Echinomycin, a quinoxaline class of cyclic peptide antibiotic, is known to bind to the VEGF-HRE sequence and inhibit VEGF expression [8]. Interestingly, echinomycin has also been reported to induce apoptosis in several types of cancer cell [9]. Therefore, targeting the HRE sequence with small molecules for a potential therapeutic option to treat cancer is possible.

## Methodology

### Sequence analysis

In homology modelling phase, we would like to look for a suitable templates to model the DNA-binding domain of HIF1, bHLH domain (both HIF-1α and HIF-1 β) sequences were aligned with structures in the protein Data Bank [10] (PDB: http://www.pdb.org/) using the NCBI-BLASTp tool [11], which is available on the NCBI website (http://www.ncbi.nlm.nlh.gov/) using a default threshold E value of 10 and an inclusion threshold value of 0.005 for the alignment between sequences of DNA-binding domain of HIF-1, bHLH domain and few homologous proteins. Multiple Sequence Alignments were created using the ClustalX tool [12].

### Construction of HIF-1 dimer by homology modeling

The 3D-model of the HIF-1 dimer was built based on template using MODELLER 9v11 [13]. The crystal structure of the PHO4 homodimer bound to DNA (1AOA) was selected as a template to model the HIF-1 dimer [14]. The sequences of the DNA-binding regions of ten bHLH-trancription factors, including PHO4, were aligned with HIF-1α and HIF-1β using ClustalX with a Gonnet weight matrix (gap opening penalty 10 and gap extension penalty 0.2) [15]. The alignment between PHO4 and HIF-1α / HIF-1β was used for model building in Modeller 9v11 [13]. To model the HIF- 1dimer, the HIF-1 subunits were modeled from the two subunits of the PHO4 homodimer. Initially hundred model objectives were generated during modeling among those one specific model objective (model structure) has been selected which has the least DOPE score energy value. The resulting HIF-1 dimer was refined by the "slow large" optimization protocol of Modeller 9v11. Computations were run on a quad core Intel 3.0 GHz Xeon X5472 processor.

### Refinement of homology model

The initial model was refined with MD simulation, which was carried out with the Visual Molecular Dynamics (VMD) tool [16]. The CHARMM 27 field [17] was used and the program NAMD [18] was used for all energy minimization and molecular dynamics (MD) simulations. All of the MD simulations were carried out in explicit water, employing periodic boundary conditions. The system was first energy minimized for 2500 runs with 50 ps and simulations for 1000000 runs with 2ns.

### Simulation parameters

The MD simulation system was equilibrated at 250 k for 10 ps with HIF-1 atoms fixed, followed by 20 ps MD without restraints. The system was subsequently simulated for 2 ns at 310 k with the following parameters. A leapfrog integrator using a time step at 1 fs integrated the classical equations of motion. The impulse based ver let-I/r-RESPA method was used perform multiple time stepping: 4 fs long-range electrostatic: 2fs for short range nonbonded forces, and 1 fs for bonded force [18]. The swift function was used to cutoff the Lennard-Jones potential, with the first cut off at 10 Å and the second cutoff at 12 Å. Short range interactions were calculated at intervals of 4 fs. All bonds involving hydrogen atoms were constrained to their equilibrium bond parameters using the SHAKE along them. Langevin dynamics were employed to maintain the pressure at 1 atm, with a Langevin pisten period of 100 fs and oscillation decay time of 50 fs. Trajectories were recorded every 200 fs. Subsequently the dynamics behavior and structural changes of the receptor was analyzed by the calculation of energy and the root mean square deviation (RMSD).

### Active site prediction

Castp Server (http://www.sts.bioe.uic.edu/castp/) was used to predict the active sites of the protein. Castp could also be used to measure area, the circumference of mouth openings of each binding site insolvent and molecular accessible surface. PDB file of protein was uploaded in the server and it showed the ligand binding sites present in protein and the site with maximum surface area and maximum surface volume was selected and all the amino acid residues involved in binding with ligands were retrieved.

### Screening Ligands

Commercially available ligands are listed in public databases, such as ZINC database, that contains more than 4.6 million compounds in ready to dock and provide 3D formats at the URL http://ZINC.dock.org/. Virtual screening has been emerged as a complementary approach to high throughput screening and has become an important in-silico technique in the pharmaceutical industry), or the more relaxed rules revised by Veber et al. 2002 [19].

In the present work, we have selected 2500 docked ligands based on structure similarity with query Chalcone natural [20]. The structure based virtual screening begins with the identification of potential ligand binding sites on the target proteins. Usually, molecules that meet the criteria for biological activity fulfill characteristics contained in the Lipinski's rule of five [21] compounds. The AutoDock Vina in PyRx Virtual Screening Tool URL http://pyrx.scripps.edu [22, 23] was used for the screening of selected ligands from Zinc database and energy minimization.

### Molecular docking studies

Docking is a computational method which predicts the preferred orientation of one molecule to a second when bound to each other to form a stable complex. Docking has been widely used to suggest the binding modes of protein inhibiters. Most docking algorithms are able to generate a large number of possible structures, thus they also require a means to score each structure to identify those that of greatest interest. Docking was performed using AutoDock Vina in PyRx Virtual Screening tool [22, 23].

PubChem and Zinc database drug molecules were docked to refined model. Lamarkian genetic algorithm was used as number of individual population (150), max number of energy evaluation (2500), max number of generation, Gene mutation rate (0.02), crossover rate (0.8), Cauchy beta (1.0) and GA window size (10.0). The grid was set whole protein due to the multi binding pocket at X=3.42, Y=-56.23, Z=98.32 and dimension AO) at X=89.92, Y=98.56, Z=98.32 and exhaustiveness 8. The pose for a given ligands identified on the basis of highest binding energy. The PyMol molecular viewer (http://www.pymol.org/) was employed to analyze the docked structures. The PyMol molecular viewer (http://www.pymol.org/) was employed to analyze the docked structures.

## Result and Discussion

### Template selection

Sequence simulating searches for both HIF-1α and HIF-2 against PDB using the NCBI-BLASTp program, revealed that the bHLH domains of both HIF-1α and HIF-12 did not present high sequence similarities with any known protein structures. The only hit from the bHLH transcription factor family was found to be the crystal structure of PHO4 (1AOA), which showed poor sequence similarities to HIF-11 (sequence identities: 27% E-value: 9, positives: 53% gaps: 5% and query coverage: 80%). The best hit was found to be the structure of the USF transcription factor- DNA complex (1AN4, sequence identities: 38%, E-value: 1x10-4. positives: 60%, gaps: 3% and query coverage: 87%). The next best hit was found to be PHO4 (sequence identities: 28%, E-value: 0.013, positives: 53%, gaps: 4% and query coverage: 95%). Hence, PHO4 and USF were used as templates for modeling HIF-1 1 and HIF-1 2 respectively.

### Homology modelling of HIF-1 dimer

We initially opted to model HIF-1 1 and HIF-12 individually using these two templates. However rigid protein-protein docking programs such as the GRAMM-X server [24] and HEX 6.1 were using to 11 and 12 docking studies .The accuracy of homology modelling depends largely on the quality of the alignment between them and template sequence. The low sequence similarity searches between the HIF-1 subunits and PHO4 could introduce errors in to the alignment. The alignment can be divided in to three regions: basic helix 1(1-30), loop (31-43) add helix2 (44-65). The alignment showed that certain residues were highly can served in their alignment. It is interesting to note that glutamate is always present at position level in these alignments, except in the case of HIF-11.Hydrophobic residues are conserved in the helix1 region with the invariable presence of L 25 and P 30.These hydrophobic residues are required for the packing of helices and P 30 is required to terminate helix 1. The loop region displays much variation and the helix 2 regions shows conserved hydrophobic residues that are required for dimerization. Both HIF-11 and HIF-12 were modeled together using different chains of PHO4 homodimer.

### Structure of HIF-1 dimer

**Figure 1:**
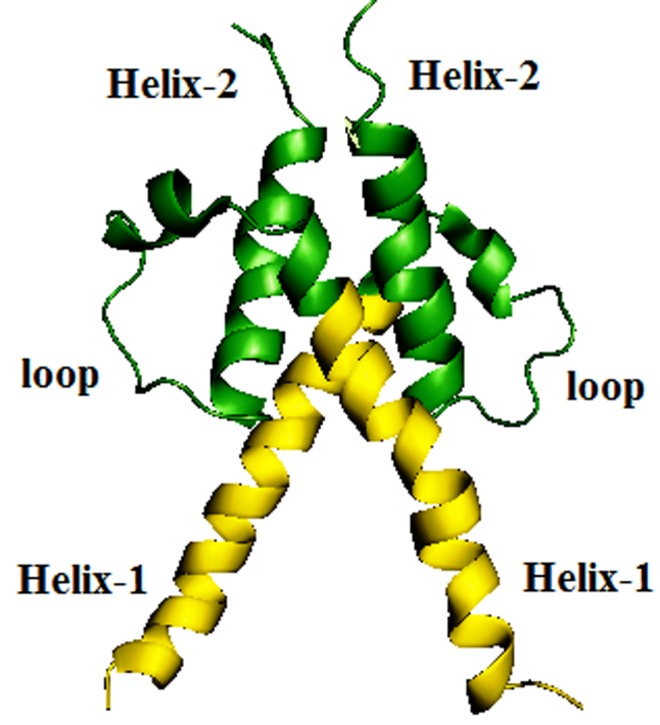
The HIF-1 model is a four-helix bundle formed by HIF- 11 and HIF-12. HIF-11 and HIF-12 HRE motif DNA binding part is highlighted with yellow color.

Each subunit has a relatively long alpha helix, rich in basic residues for DNA binding and a shorter helix. These two helices connected by a long loop containing a shot turn of a helix that makes the loop compact. The loop determines the directionality of the two helices. The dimer is a four-helix bundle with a packed hydrophobic interior. The second helix is very short, which might affect the tight HIF-1 complex formation. However, the PIS domains form the respective sub units of HIF-1 dimer to give additional support to the complex (Figure 1).

The structure quality of HIF-1 was assessed using PROCHECK server. The structure was found to have (92.5%) of its residues in the most favored regions and the remaining (7.5%) of its residues in additionally allowed regions in the Ramachandran plot, these suggesting that model is of good quality. Using PyMol molecular viewer performed the superimposition of model with template. This model was used for docking studies with screened compounds.

### Screening Ligands

Virtual screening is a proficient approach in discovering inhibitors with novel chemical scaffolds. Two-dimensional structure of gossypol was used as query to search for similar compounds in the Zinc database. Then, approximately 2500 compounds were screened, and the all compounds were saved for further molecular docking. Attempts to screening of Chalcone like compounds, that is cytotoxic at high doses, have produced several compounds retaining activity against both the target enzyme.

### Docking Studies

Processing of the HIF-1 dimer included energy minimized and molecular dynamics simulations. The refinement of structure of protein was used for the dock. AutoDock Vina was used for the docking studies. The docked conformation corresponding to the lowest binding energy was selected as the most probable binding conformation. The total screened 2500 compounds were docked into the active site of HIF-1 dimer. The best ten zinc compounds showed high binding energies and significant affinities with target protein of HIF-1 dimer the values are represented in Table 1. Which all the ligands were embedded within the active site of target protein were observed forming hydrogen bonds with it position as Chalcone established active site of target protein. The best docked compounds such as Zinc04280532, Zinc04280533, Zinc04280469, Zinc04280534, Zinc16405915, Zinc04277060, Zinc04280538, Zinc04582923, Zinc05280554 and Zinc05943723 were found to be shown highest binding energies viz., -11.9, - 10.5, -9.5, -8.9, -8.3, -8.1, -8.1, -7.6, -7.5, -7.2 and -6.6 kcal/mol respectively Table 1.

**Figure 2:**
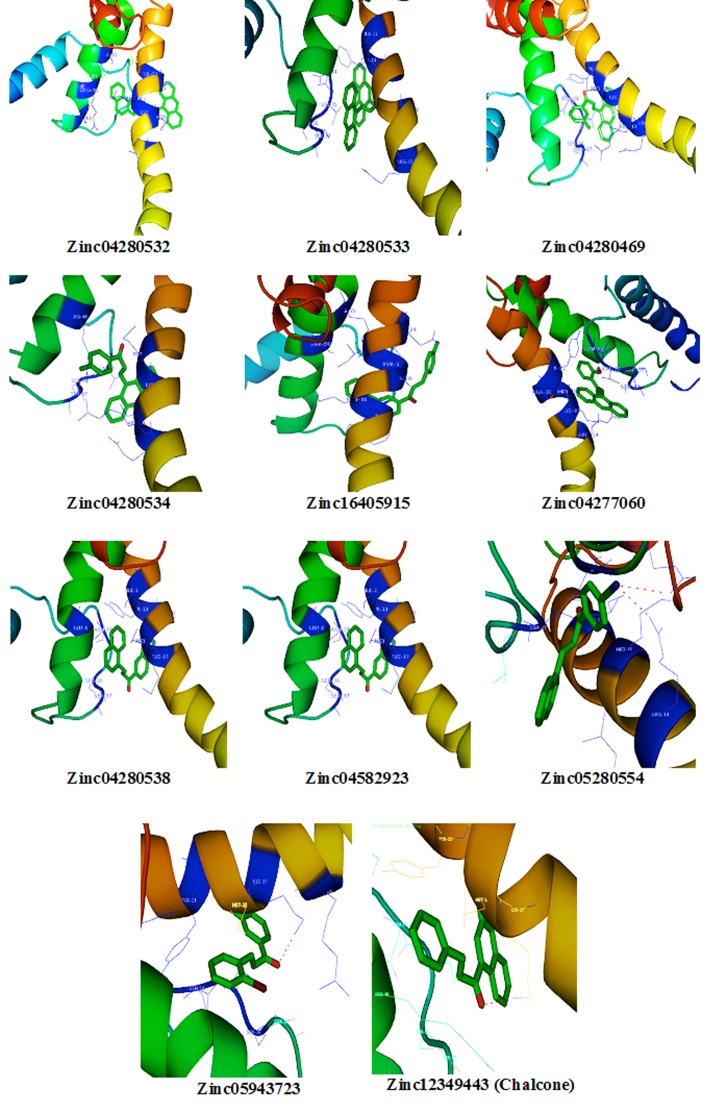
Graphical representation of HIF-1protein and Chalcone and its analogues interactions.

Hydrogen bonds play a role in stabilizing the protein-ligand complex. The Zinc database compounds also exhibit several hydrogen bonding moieties the best binding affinity compounds were obtained through the molecular docking studies. The obtained compounds were binding with active site of target protein. The active site of amino acids plays a key role, to interact the hypoxia responsive element (HRE) present in the promoter region of hypoxia responsive genes. The compounds Zinc4280532, Zinc04280533, Zinc04280534, Zinc04277060, Zinc04280538, Zinc04582923, Zinc05280554 and Chalcone compounds were bound with the binding affinity by the formation of hydrophobic interactions such as Van der Waal and electrostatic interactions with in the active site of HIF-1 dimer. The compound Zinc4280532 was bound with the binding affinity -11.9 kcal/mol by the electrostatic interactions with TYR-21, ASN-34, VAL-35, MET-18, LYS-17, and SER-36 in Helix-1 region of HIF-1 dimer. Zinc04280533 was bound with the binding affinity -10.5 kcal/mol by the electrostatic interactions with TYR- 21, ASN-34, VAL-35, MET-18, LYS-17, SER-36 in of helix-2 and helix-1 of HIF-1 dimer. Zinc04280469 was bound with the binding affinity -9.5 kcal/mol by the formation of one hydrogen bond with LYS17 residue of HIF-1 dimer protein. Zinc04280534 was bound with the binding affinity -8.9 kcal/mol by the electrostatic interactions with TYR-21, ASN-34, VAL-35, MET-18, LYS-17, SER-36 in of helix-2 and helix-1 of HIF-1 dimer. Zinc16405915 was bound with the binding affinity -8.3 kcal/mol by the formation of two hydrogen bonds with ARG46 and ARG 14 residue of HIF-1 dimer protein. Zinc04277060 was bound with the binding affinity -8.1 kcal/mol by the electrostatic interactions with LYS-17, TYR-21, VAL-35, ASN-34, ARG-55, LEU-47 in of helix-2 and helix-1 of HIF-1 dimer. Zinc04280538 was bound with the binding affinity -8.1 kcal/mol by the electrostatic interactions with TYR-21, ASN-34, MET-38, VAL-35 in of helix-2 and helix-1 of HIF-1 dimer. Zinc04582923 was bound with the binding affinity -7.6 k.cal/mol by the electrostatic interactions with ASN- 34, MET-18, LYS-17, SER-36, SER-37, VAL-35 in of helix-2 and helix-1 of HIF-1 dimer. Zinc05280554 was bound with the binding affinity -7.5 k.cal/mol by the electrostatic interactions with ASN- 34, VAL-35, MET-18, LYS-17, SER-36, SER-37 in of helix-2 and helix-1 of HIF-1 dimer. Zinc05943723 was bound with the binding affinity -7.2 k.cal/mol by the formation of two hydrogen bonds with ARG46 and ARG 14 residue of HIF-1 dimer protein. Zinc12349443 (Chalcone) was bound with the binding affinity -6.6 k.cal/mol by the electrostatic interactions with MET-18, LYS-17, VAL-35, SER-36, SER-37 in of helix-2 and helix-1 of HIF-1 dimer. The protein and ligand interactions showed that active site amino acids play a key role in bound to the best compounds, may be these amino acids are involve for inhibitory action of HIF-1 protein. The lead compounds and their interactions with active site of residues are graphically represented in Figure 2.

The lead hit compounds satisfied the Lipinski's rule of five with zero violations and also the octanol/water partition coefficient (miLogp), a useful parameter for predicting the drug transport properties like absorption, bioavailability, permeability and penetration. As well as topological molecular polar surface area (TPSA), number of atoms, their molecular weight (MW), number of hydrogen donors and number of hydrogen acceptors. Topological parameters are number of rotatable bonds and it describes the molecular flexibility of these compounds. All the values of best binding affinity compounds were shown in Table 2. Our investigations revealed that the selected compounds have exhibited significant binding affinities within the active site of HIF-1 dimer, when compare to query compound Chalcone. Based upon this study, these compounds may be used as leads for developing an effective anti angiogenesis drugs.

## Conclusion

The aim of the present study was to explore new inhibitors and to investigate the role of binding cavities of HIF-1 dimer in angiogenesis by the structure based virtual screening and molecular docking studies. Protein structure refinement by 2ns molecular simulation and energy minimizations improved the general structure of protein and stable RSMD. The screened, ten compounds Zinc04280532, Zinc04280533, Zinc04280469, Zinc04280534, Zinc16405915, Zinc04277060, Zinc04280538, Zinc04582923, Zinc05280554 and Zinc05943723 were found to be more binding than Chalcone. This class of chemicals has been developed in an attempt to reduce the toxicity. All of the compounds we discovered in this work bind to their respective binding sites by creating hydrogen bonds and hydrophobic interactions with important residues in the binding pockets. We performed a detailed analysis of the atomic interactions between each potential compound and residues inside the binding site to identify residues interact with the compounds. In conclusion, the present findings identify these lead compounds as major inhibitors of HIF-1 dimer protein.

## Conflicts of interests

The authors declare no conflict of interest.

## Figures and Tables

**Table 1:** Chalcone and Chalcone -analogue compounds along with their respective interaction affinities and their surrounding residues.

Rank	Zinc ID's	Binding affinity	Interactions (Protein-----Ligand)	No. of Hydrogen Bonds	Bond angle (degree)	Bond-length (Å)
1	Zinc04280532	-11.9	TYR-21, ASN-34, VAL-35, MET-18, LYS-17, SER-36	0	-	-
2	Zinc04280533	-10.5	TYR-21, ASN-34, VAL-35, MET-18, LYS-17, SER-36	0	-	-
3	Zinc04280469	-9.5	LYS17CN----O24C15	1	104.1	3.1
4	Zinc04280534	-8.9	VAL-35, ASN-34, SER-36, MET-18, LYS-17	0	-	-
5	Zinc16405915	-8.3	1.ARG46CN----N1C5	2	121.9	3.5
			2.ARG14CN----N1C5		155	2.9
6	Zinc04277060	-8.1	LYS-17, TYR-21, VAL-35, ASN-34, ARG-55, LEU-47	0	-	-
7	Zinc04280538	-8.1	TYR-21, ASN-34, MET-38, VAL-35	0	-	-
8	Zinc04582923	-7.6	ASN-34, MET-18, LYS-17, SER-36, SER-37, VAL-35	0	-	-
9	Zinc05280554	-7.5	ASN-34, VAL-35, MET-18, LYS-17, SER-36, SER-37	0	-	-
10	Zinc05943723	-7.2	1.ARG46CN----N1C6	2	122	3.5
			2.ARG14CN----N1C6		155.6	2.9
11	Zinc12349443 (Chalcone)	-6.6	MET-18, LYS-17, VAL-35, SER-36, SER-37	0	-	-

**Table 2:** In-silico ADMET prediction by OSIRIS property explorer and Lipinski 'Rule of 5' by Molinspiration servers.

Zinc Id	Mutagenicity	Tumorigenic	Irritant	Reproductive effect	C log p	Solubility	MW	No. of H-acceptor	No. of H-donors	Rotator bonds	Drug liking	Drug score
Zinc04280532	+	+	-	-	8.24	10.26	408	1	0	3	-3.36	0.06
Zinc04280533	+	-	-	-	8.24	10.26	408	1	0	3	1.82	0.08
Zinc04280469	+	-	-	-	6.65	-6.9	306	1	0	3	-5	0.09
Zinc04280534	+	-	-	-	6.19	-7.4	322	1	0	3	-3.24	0.1
Zinc04277060	+	-	-	-	5.93	-7.37	326	1	0	3	-0.32	0.14
Zinc04280538	+	-	-	-	6.48	-7.79	342	1	0	3	-0.25	0.13
Zinc16405915	+	-	-	-	5	-5.79	272	2	0	3	-0.09	0.21
Zinc04582923	-	-	-	-	5.49	-6.38	326	2	1	3	-4.92	0.2
Zinc05943723	+	-	-	-	3.2	-5.07	392	4	0	6	-0.67	0.25
Zinc05280554	+	+	+	+	4.46	-4.78	391	6	3	9	1.7	0.12
Zinc06339475	+	-	-	-	5.39	-6.28	336	1	0	3	-5.43	0.11
Zinc04280535	+	-	-	-	6.19	-7.4	322	1	0	3	-1.8	0.11
